# Identification of New Anti‐*Trypanosoma brucei* Leads: An Integrated CRK12‐Guided Virtual Screen With Experimental Validation

**DOI:** 10.1111/cbdd.70389

**Published:** 2026-08-30

**Authors:** Qin Li, Chenggong Fu, Jiayi Luo, Qianqian Zhang, Lingling Wang, Xiaomeng Liu, Jiayue Qiu, Xiaoqing Gong, Henry H. Y. Tong, Xiaojun Yao, Dehua Lai, Huanxiang Liu

**Affiliations:** ^1^ Faculty of Applied Sciences Macao Polytechnic University Macao SAR China; ^2^ MOE Key Laboratory of Gene Function and Regulation, State Key Laboratory of Biocontrol and Guangdong Provincial Key Laboratory of Aquatic Economic Animals, School of Life Sciences Sun Yat‐Sen University Guangzhou China

**Keywords:** CRK12, drug discovery, human African trypanosomiasis (HAT), molecular docking, neglected tropical disease, virtual screening

## Abstract

Human African trypanosomiasis (HAT), caused by *Trypanosoma brucei*, remains a neglected tropical disease with a critical shortage of therapeutic options, underscoring the need for new chemotypes. Cell division cycle‐2‐related kinase 12 (CRK12) has emerged as a genetically essential and chemically validated target in kinetoplastids, yet experimentally supported CRK12‐targeting chemotypes against *T. brucei* remain limited, and no experimental CRK12 structure is available. Here, we developed an integrated computational‐experimental screening workflow to accelerate CRK12‐guided hit discovery, combining ligand‐based prescreening, structure‐based virtual screening, molecular dynamics (MD) refinement, and experimental validation. An 8.6‐million‐compound library was prescreened using MACCS fingerprint screening and complex‐based pharmacophore screening, followed by staged docking and MD simulations to prioritize candidates for experimental testing. Notably, four of ten purchased compounds showed strong growth inhibition against *T. brucei* at 10 μM, with IC_50_ values of 6.09, 1.47, 0.81, and 1.33 μM, demonstrating a promising hit rate for this data‐limited target. Binding‐mode analysis revealed a conserved hinge‐anchoring interaction pattern in the ATP‐binding pocket, providing a structural rationale for follow‐up analogue design. Overall, this study identifies new chemical starting points for anti‐ *T. brucei* drug discovery and demonstrates the utility of an AlphaFold2‐enabled CRK12‐guided screening strategy for targets with limited structural and ligand data.

## Graphical abstract

An integrated computational‐experimental workflow enabled CRK12‐guided hit discovery against Trypanosoma brucei under limited structural and ligand data. The workflow of virtual screening and anti‐ *T. brucei* bioassays identified 4 hit compounds, including three low‐micromolar leads (IC_50_ = 1.47, 0.81, and 1.33 μM).

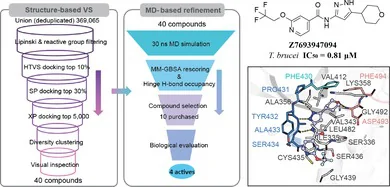

## 1 Introduction

Human African trypanosomiasis (HAT), caused by *Trypanosoma brucei* and transmitted by tsetse flies, remains a neglected tropical disease and continues to impose a health and socioeconomic burden in endemic regions (WHO 2023). The disease comprises a chronic gambiense form that accounts for most reported cases and a less frequent but more acute rhodesiense form (Lejon et al. 2025; WHO 2023). HAT follows a two‐stage clinical course, an early haemolymphatic phase followed by a meningoencephalitic phase in which parasites enter the central nervous system, leading to neurological manifestations and death without treatment (De Rycker et al. 2023). Although reported cases have declined over the past two and a half decades, substantial populations remain exposed, with 41.5 million people living in areas at risk during 2018–2022 (Franco et al. 2024).

Control of HAT relies on case detection, treatment, and vector control (Lejon et al. 2025; WHO 2023). Despite this progress, available therapies continue to be limited by stage‐dependent administration, operational challenges, and safety constraints (Field et al. 2017; Lejon et al. 2025). Historical drugs, including arsenicals used for late‐stage disease, are associated with severe toxicity and often require hospitalization of patients and prolonged administration (Seixas et al. 2020). Recent therapeutic advances have improved access, including the introduction of oral fexinidazole for gambiense‐HAT, which has been registered for treating both disease stages and is also recommended for rhodesiense‐HAT (DNDi 2026a, 2026b). Acoziborole has also shown promising results as an oral single‐dose candidate in a Phase 2/3 clinical trial (Betu Kumeso et al. 2023). However, eligibility and safety constraints remain for specific patient groups. For instance, fexinidazole is not recommended for severely ill patients or for young children and must be administered by trained health professionals (Lindner et al. 2025; Lindner et al. 2020). Given that only a small number of approved drugs are currently available, the risk of resistance emergence and the need for additional chemotypes with distinct mechanisms of action remain pressing concerns (Koning 2020; Lejon et al. 2025).

Antiparasitic drug discovery has primarily focused on essential proteins, such as cell cycle regulators, membrane proteins, and mitochondrial structural proteins (Field and Carrington 2009; Li 2012; Pereira et al. 2020). In this context, cell division cycle‐2‐related kinase 12 (CRK12), a kinase involved in parasite cell‐cycle regulation, has emerged as a compelling target supported by genetic and chemical validation. In *T. brucei* , CRK12 is essential for parasite survival, and its depletion results in enlargement of the flagellar pocket and defects in endocytosis (Monnerat et al. 2013). In *Leishmania*, pyrazolopyrimidines such as GSK3186899/DDD853651 provided strong evidence linking antiparasitic activity to CRK12 inhibition (Wyllie et al. 2018). More directly relevant to African trypanosomes, a diaminothiazole series exemplified by cmpd2 was confirmed to target CRK12 through mode‐of‐action studies and validation of resistance‐associated CRK12 mutations (Altmann et al. 2022; Smith et al. 2022). Cmpd2 showed potent whole‐cell activity against *T. brucei* , with a reported EC_50_ of 0.96 nM (Altmann et al. 2022). Despite these advances, experimentally supported CRK12 chemotypes against *T. brucei* remain limited, leaving clear scope to expand scaffold diversity for antitrypanosomal discovery. This gap, together with the absence of an experimentally resolved CRK12 structure, motivates the development of structure‐guided computational strategies capable of prioritizing compounds for experimental testing.

Computer‐aided drug design (CADD) provides a practical framework for triaging large chemical libraries and computationally enriching candidates suitable for experimental follow‐up (Bender et al. 2021; Yu and MacKerell Jr. 2017). Virtual screening (VS) is widely applied through complementary ligand‐based and structure‐based routes. Ligand‐based approaches, such as fingerprint similarity (Hutter 2022) and pharmacophore modeling (de Souza et al. 2017; Yang et al. 2026), can rapidly enrich for candidates consistent with known chemotypes or interaction features, whereas structure‐based docking uses three‐dimensional binding‐site information to rank putative binders. However, docking‐based prioritization is limited by scoring‐function accuracy and incomplete sampling of receptor conformations, and treating receptors as rigid can be particularly problematic when docking to apo conformations or computationally predicted structures (Yuriev et al. 2011). Molecular dynamics (MD) can therefore complement docking by providing a physics‐based assessment of pose stability and interaction persistence, thereby supporting more reliable prioritization beyond docking scores alone.

**FIGURE 1:**
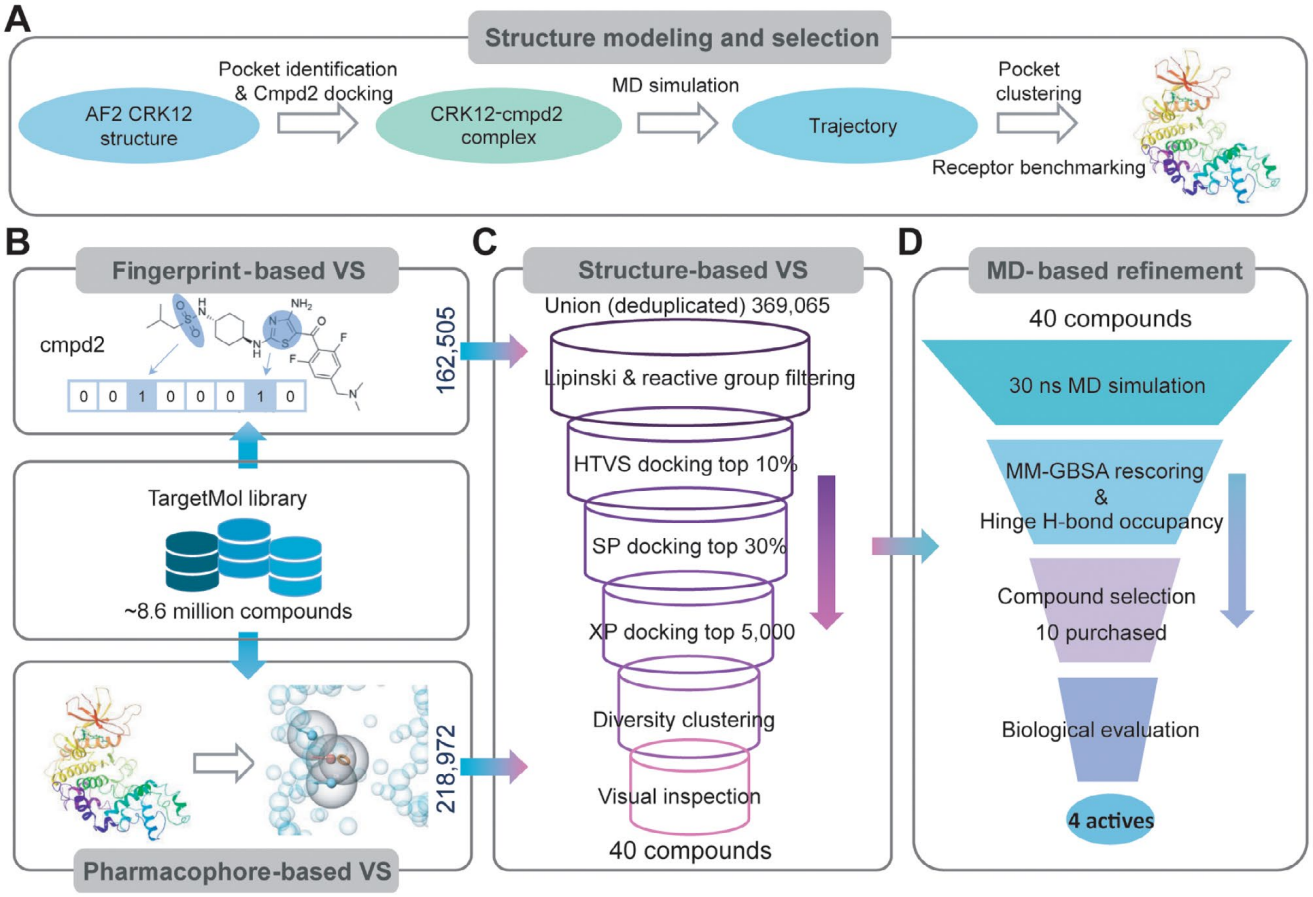
Overview of the integrated virtual screening and validation workflow for CRK12‐guided anti‐ *T. brucei* hit discovery. (A) Receptor modeling and selection. An AlphaFold2‐derived CRK12 model was refined by long‐timescale MD simulation in complex with cmpd2, followed by pocket‐focused clustering and retrospective benchmarking to select the receptor conformation for docking. (B) Ligand‐based prescreening and chemical structure of cmpd2. The TargetMol library containing approximately 8.6 million compounds was screened by MACCS fingerprint and complex‐based pharmacophore approaches, yielding 162,505 MACCS fingerprint hits and 218,972 pharmacophore‐selected compounds, respectively. Their union contained 369,065 unique compounds after deduplication. (C) Structure‐based virtual screening. The union set was subjected to drug‐likeness filtering and reactive‐group removal, followed by staged docking using Glide HTVS, SP, and XP modes. Diversity clustering and visual inspection were then used to select 40 compounds for MD‐based refinement. (D) MD‐based refinement and biological testing. The 40 CRK12‐ligand complexes were subjected to 30 ns MD simulations, MM‐GBSA rescoring, and hinge hydrogen bond occupancy analysis. Ten compounds were subsequently purchased for in vitro testing, and four showed anti‐ *T. brucei* activity.

To enable CRK12‐guided screening of a multi‐million‐compound library under conditions of limited ligand and structural data, we designed a stepwise workflow integrating ligand‐based prescreening, structure‐based docking, MD‐based refinement, and experimental validation (Figure 1). Given its potent whole‐cell activity against *T. brucei* and the experimental evidence supporting CRK12 as its molecular target, cmpd2 was selected as the query ligand for fingerprint‐based screening and as the reference ligand for CRK12‐cmpd2 complex refinement and complex‐based pharmacophore construction. Its chemical structure is shown in Figure 1B. Although a single query ligand cannot represent the full chemical diversity of potential CRK12‐targeting compounds, fingerprint‐based screening was complemented by pharmacophore screening and subsequent structure‐based screening. An MD‐refined CRK12‐cmpd2 conformational ensemble was first subjected to pocket‐focused clustering and retrospective active/decoy benchmarking to select a receptor conformation suitable for prospective docking. The TargetMol library was then screened using two complementary ligand‐based approaches. MACCS fingerprint screening was used to retrieve compounds sharing predefined substructural features with cmpd2, whereas complex‐based pharmacophore screening was used to identify compounds satisfying the three‐dimensional interaction features derived from the representative CRK12‐cmpd2 binding pose. Because these approaches encode different aspects of ligand recognition, their union was retained to preserve candidates identified by either prescreening strategy.

The resulting prescreened set was subsequently filtered and processed through staged docking using Glide HTVS, SP, and XP modes. This hierarchical design allowed rapid screening at the initial stage while reserving the more computationally demanding docking protocols for progressively smaller subsets. Diversity clustering and visual inspection were then used to reduce structural redundancy and exclude compounds with unfavorable binding poses. The selected CRK12‐ligand complexes were further evaluated using MD simulations, MM‐GBSA analysis, and hinge hydrogen bond occupancy analysis to assess pose stability, relative binding energy, and persistence of hinge‐region interactions. Finally, the prioritized compounds were tested against bloodstream form *T. brucei* . Through this sequential workflow, each stage contributed a distinct selection criterion and progressively reduced the initial library to a tractable set for experimental evaluation. Among the 10 purchased compounds, four showed strong growth inhibition at 10 μM and were confirmed as active, with IC_50_ values ranging from 0.81 to 6.09 μM.

## 2 Results and Discussion

### 2.1 Fingerprint‐Based Similarity Screening

**FIGURE 2:**
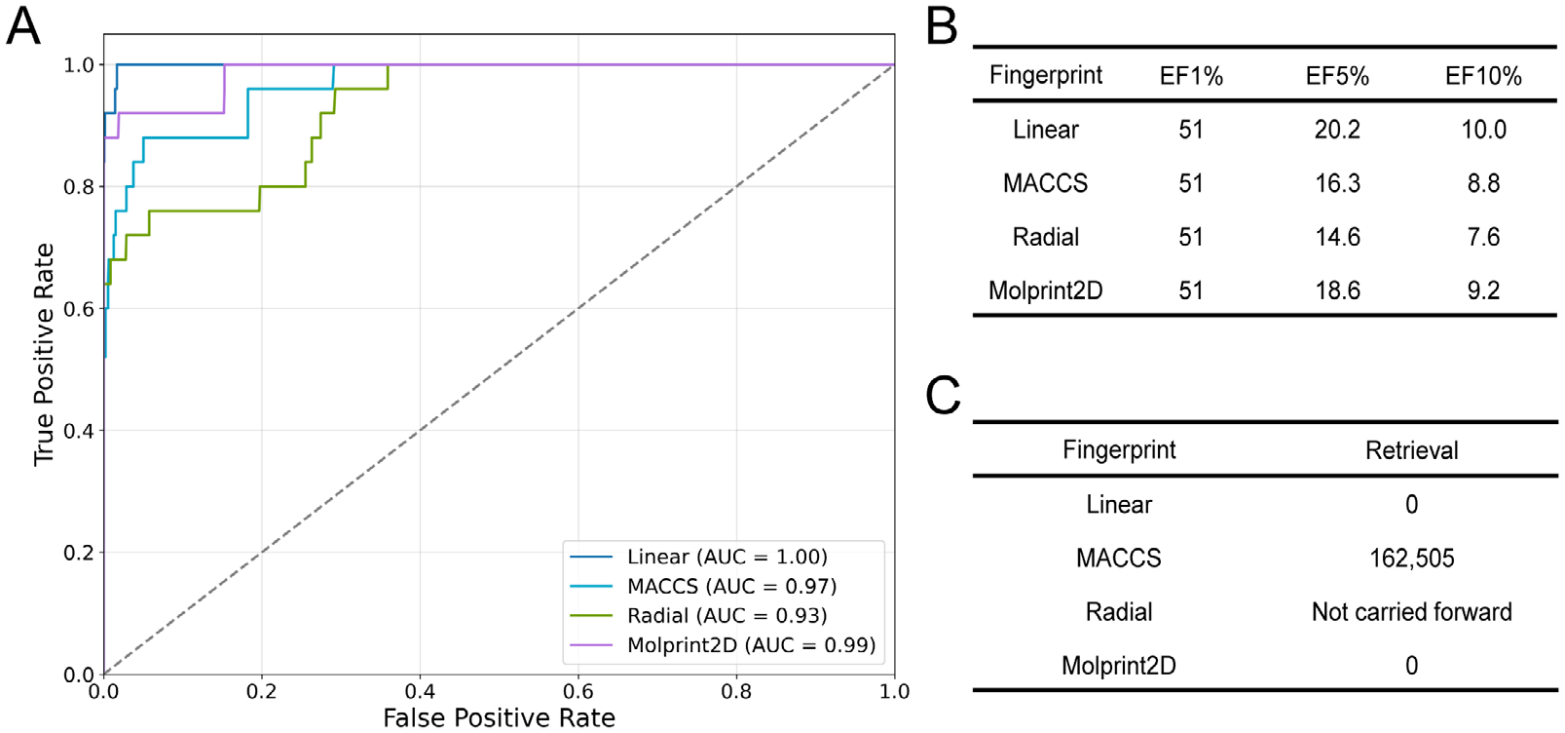
Benchmark performance and library retrieval of fingerprint‐based screening. (A) ROC curves obtained by ranking the CRK12 active/decoy benchmark by Tanimoto similarity to the reference ligand cmpd2 using Linear, MACCS, Radial, and MolPrint2D fingerprints. AUC values are shown in the legend. (B) Early enrichment factors computed on the benchmark at 1%, 5%, and 10% of the ranked list. (C) Numbers of compounds retrieved from the TargetMol library containing ~8.6 million compounds at Tanimoto ≥ 0.60 using each fingerprint.

To select a fingerprint for prospective screening, we first evaluated four commonly used fingerprints using the established CRK12 active/decoy benchmark and then compared the numbers of compounds retrieved from the TargetMol library at a fixed similarity threshold. On the benchmark ranking task, all fingerprints separated actives from decoys well, with ROC‐AUC values ranging from 0.93 to 1.00. Linear and MolPrint2D showed the strongest overall discrimination, with AUC values of 1.00 and 0.99, followed by MACCS at 0.97 and Radial at 0.93 (Figure 2A). Early enrichment was also high for all encodings. The EF1% values were identical across the four fingerprints, whereas EF5% and EF10% provided clearer differentiation, with Linear and MolPrint2D showing higher enrichment than MACCS and Radial at broader early cutoffs (Figure 2B). This pattern indicates that, under the current benchmark composition, extremely early cutoffs capture a similar fraction of actives across fingerprints, while differences become more apparent as the early selection window expands. We then tested prospective retrieval from the TargetMol library using a fixed Tanimoto threshold of 0.60. Notably, Linear and MolPrint2D retrieved no compounds at this cutoff, whereas MACCS returned 162,505 compounds (Figure 2C). Radial was not carried forward because it showed the weakest benchmark performance among the tested fingerprints. The divergent retrieval behavior is consistent with the information content of the encodings. Linear and MolPrint2D fingerprints emphasize the atomic environments and higher‐resolution structural detail, so a threshold of 0.60 selects only very close analogs. The absence of hits suggests that near neighbors of cmpd2 are scarce in the compound library at this similarity level. In contrast, MACCS keys encode the presence of predefined substructures and can capture motif‐level resemblance even when close analogs are uncommon, resulting in a larger hit set at the same cutoff. Therefore, MACCS fingerprint screening was used at the prescreen stage to reduce the library before structure‐based screening.

### 2.2 Complex‐Based Pharmacophore Screening

**FIGURE 3:**
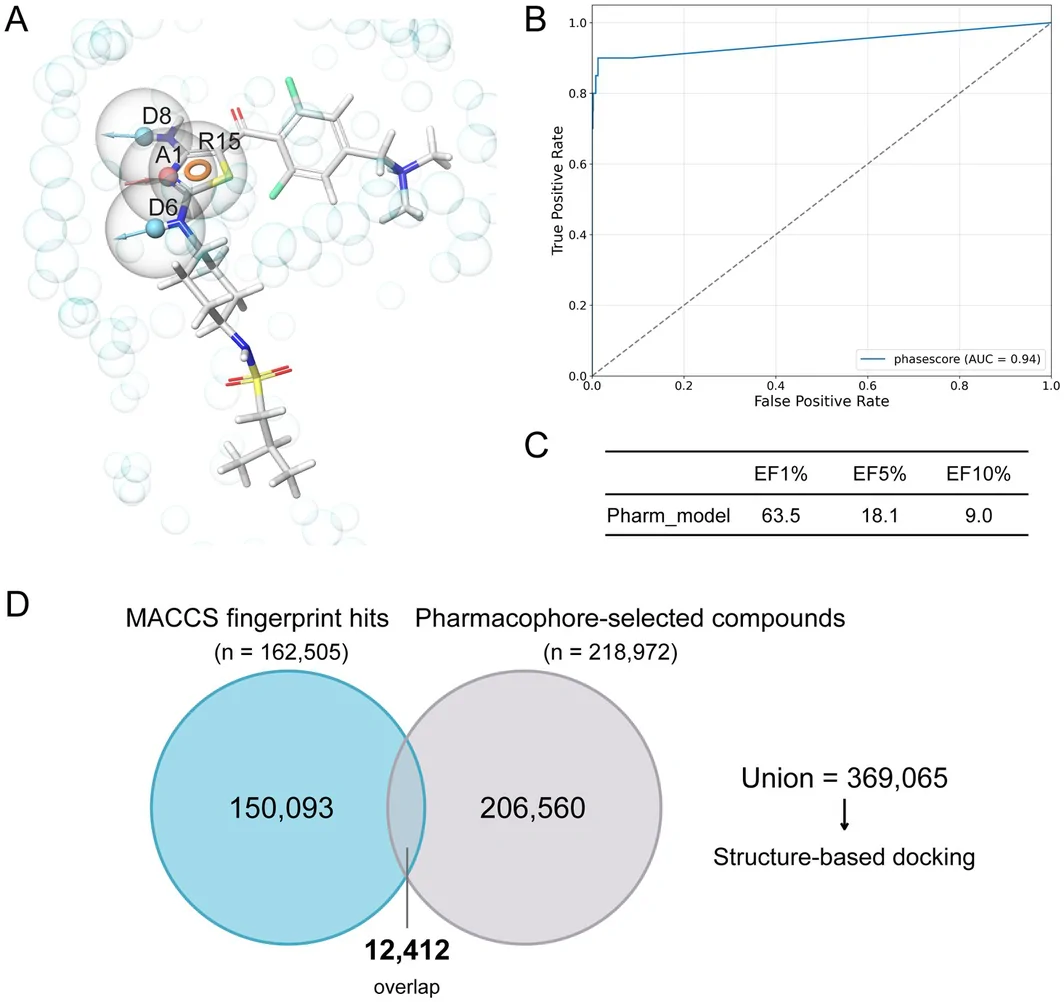
Complex‐based pharmacophore model, retrospective performance, and integration with MACCS fingerprint screening. (A) The ADDR pharmacophore hypothesis derived from a representative CRK12‐cmpd2 complex. The ligand is shown as white sticks. The aromatic ring feature is shown in orange, hydrogen bond donor features are shown in blue, the hydrogen bond acceptor feature is shown in pink, and excluded‐volume spheres are shown in cyan. (B) ROC curve for ranking the benchmark by Phase score, with the corresponding AUC. (C) Early enrichment factors at 1%, 5%, and 10% of the ranked list obtained with the pharmacophore model. (D) Overlap of compounds identified by MACCS fingerprint screening and pharmacophore screening, with the union set retained for subsequent structure‐based docking.

To complement fingerprint‐based screening, we applied complex‐based pharmacophore screening. Pharmacophore screening focuses on key interaction features and can therefore retrieve candidates that do not necessarily closely resemble the reference scaffold. The pharmacophore model was derived from a representative CRK12‐cmpd2 complex from the MD ensemble and defined as a four‐feature hypothesis, ADDR, comprising one hydrogen bond acceptor (A), two hydrogen bond donors (D), and one aromatic ring feature (R) (Figure 3A). Retrospective evaluation on the CRK12 active/decoy benchmark showed good performance, with an AUC of 0.94 and enrichment factors of 63.5 at 1%, 18.1 at 5%, and 9.0 at 10% (Figure 3B,C). We then applied this pharmacophore model to screen the 8.6‐million‐compound TargetMol library. The top 300,000 matched poses were retained according to Phase score and deduplicated by compound name, yielding 218,972 unique compounds for downstream screening. Comparison of the two prescreening outputs revealed that only 12,412 compounds overlapped between the 162,505 MACCS fingerprint hits and the 218,972 pharmacophore‐selected compounds (Figure 3D). This modest overlap reflects the fundamentally different selection criteria of the two approaches: MACCS‐based screening prioritizes two‐dimensional substructural similarity to the query ligand cmpd2, whereas pharmacophore screening relies on the three‐dimensional spatial arrangement of interaction features. To avoid premature filtering and preserve chemical diversity, we therefore retained the union of the two sets rather than the intersection, yielding 369,065 unique compounds for subsequent structure‐based docking. This union‐based strategy ensured that candidates identified by either prescreening criterion were carried forward to the more stringent docking stages, preventing overly restrictive filtering at this early step.

### 2.3 Structure‐Based Virtual Screening

To select a receptor conformation for structure‐based docking, we benchmarked the MD‐refined receptor conformation used previously, termed Refined (Li et al. 2025), and the five most populated pocket‐cluster centroids (Rep C0‐C4) on the established benchmark. This comparison was carried out because no experimentally determined *T. brucei* CRK12 structure is available, and docking performance depends strongly on the pocket conformation. Docking was performed with Glide in SP and XP modes, and performance was assessed using ROC‐AUC and enrichment factors at 1%, 5%, and 10% of the ranked list (Table 1). In Glide SP docking, Refined achieved the highest ROC‐AUC of 0.98, indicating the strongest overall separation across the ranked list. Rep C0 showed a very similar AUC of 0.97 but improved early enrichment, with EF1% increasing from 39 for Refined to 49 for Rep C0. Rep C1 and Rep C2 reached the highest EF1% values in SP, both at 54, yet their AUC values dropped to 0.92, suggesting that these conformations can place some actives very early but provide weaker global ranking across the full list. Rep C3 retained good discrimination with an AUC of 0.96 but exhibited a low EF1% of 19, indicating poor early recognition despite acceptable overall separation. Rep C4 performed worst in SP with an AUC of 0.86 and reduced early enrichment. In Glide XP docking, Rep C0 provided the most balanced profile. It achieved an AUC of 0.99 together with the strongest early enrichment among all receptors, with EF1%, EF5%, and EF10% values of 44, 19, and 10, respectively. Refined maintained an AUC of 0.98 but showed weaker early enrichment in XP, with EF1% of 24. Rep C4 reached an AUC of 0.99 in XP but had lower enrichment factors than Rep C0, indicating that high AUC alone does not guarantee strong early retrieval. Rep C2 matched Rep C0 in EF1% under XP but showed a lower AUC of 0.96 and reduced EF5% and EF10%, consistent with less stable ranking beyond the earliest selection. These results show that the receptor choice is sensitive to binding‐site conformation and indicate that receptor‐conformation selection should consider both global discrimination and early enrichment. On this basis, Rep C0 was selected for prospective screening because it maintained one of the highest AUC values and consistently strong enrichment, particularly under the more stringent XP protocol. The observation that the most populated pocket state also yielded the best overall screening profile supports the use of pocket‐focused clustering as a practical strategy to derive effective receptor conformations for structure‐based virtual screening.

**TABLE 1:** Retrospective docking performance of Refined and Rep C0‐C4 on the CRK12 active/decoy benchmark.

Receptor conformation (cluster centroid)	Glide SP				Glide XP			
	AUC	EF1%	EF5%	EF10%	AUC	EF1%	EF5%	EF10%
Refined (previous work)	**0.98**	39	**17**	**9**	0.98	24	16	9.5
Rep C0 (49.6%)	0.97	49	15	8.5	**0.99**	**44**	**19**	**10**
Rep C1 (35.3%)	0.92	**54**	15	8.5	0.98	39	17	9.5
Rep C2 (7.1%)	0.92	**54**	16	8	0.96	**44**	14	8.5
Rep C3 (3.9%)	0.96	19	13	**9**	0.98	20	15	9.5
Rep C4 (2.8%)	0.86	39	13	6.5	**0.99**	34	16	8

*Note:* ROC‐AUC and EF1%, EF5%, and EF10% are reported for Glide SP and XP. Cluster population is reported for each centroid. Bold values indicate the highest value for each metric within each docking protocol, with tied values also shown in bold.

Following receptor selection, we implemented a staged docking workflow using Glide to prioritize compounds for downstream MD‐based refinement and experimental testing. The union set was first subjected to Lipinski's rule‐of‐five filtering and reactive‐group removal. The filtered library was then advanced through the staged docking workflow. HTVS was used for the initial docking, and the top 10% of ranked compounds were retained and redocked using Glide SP. The top 30% from SP were then advanced to XP docking. The top 5000 XP‐ranked compounds were clustered into 100 groups to reduce structural redundancy. Representative compounds were selected based on docking rank, cluster representation, and visual inspection of the predicted binding modes, with attention to hinge‐region hydrogen bonding and the absence of severe steric clashes. This procedure yielded 40 compounds for subsequent MD‐based refinement.

### 2.4 MD‐Based Refinement and MM‐GBSA Binding Free Energy Estimation

The 40 CRK12‐ligand complexes selected after structure‐based screening were subjected to 30 ns MD simulations, and MM‐GBSA binding free‐energy estimates were calculated from snapshots extracted from the final 5 ns of each trajectory. In parallel, hinge interaction stability was evaluated by monitoring the occupancy of hydrogen bonds involving the CRK12 hinge residue ALA433 during the simulations. The RMSD results of the binding‐site pocket and ligand heavy atoms across all 40 systems are shown in Figure S1. Pocket and ligand RMSDs were generally stable, with occasional increases in ligand RMSD observed for a small subset of complexes. Final compound prioritization was based primarily on the ranking of MM‐GBSA binding free‐energy estimates, with ALA433 hydrogen bond occupancy used as a complementary measure of hinge‐interaction persistence. Practical procurement constraints limited the number of compounds that could be advanced to biological testing. Accordingly, the 11 highest‐ranked compounds were prioritized for purchase. One compound was not commercially available, and the remaining 10 compounds were purchased for activity testing. The physicochemical properties, Glide XP docking scores, and MD‐derived metrics of the 10 purchased compounds are summarized in Table 2. The computational metrics and selection status of all 40 compounds are provided in Table S1. The purchased compounds showed consistently high occupancies of hydrogen bonds involving ALA433, ranging from 92.8% to 99.6%, supporting persistent hinge interactions during the simulations. The corresponding MM‐GBSA energy components and hinge hydrogen‐bond details for the purchased compounds are reported in Tables S2 and S3. These MD‐based analyses complemented the docking‐stage prioritization and supported the final selection of compounds for experimental testing.

**TABLE 2:** Physicochemical properties (QikProp), Glide XP docking scores, and MD‐derived metrics for the 10 purchased compounds advanced to experimental testing.

Vendor ID	Internal ID	MW	QPlogPo/w	QPlogS	donorHB	accptHB	PSA	Glide XP Docking score	$∆G_{\text{bind}}$	Hinge H‐bond occupancy (%)
Z4518256454	7431599	364.36	2.85	−5.77	2	5.25	143.81	−11.41	−44.82	98.8
Z1275018845	560407	367.45	3.21	−6.00	3	5.5	111.63	−9.12	−43.57	92.8
Z1232077491	1206270	415.85	1.87	−5.12	3.5	9.5	128.84	−10.65	−43.11	97.6
Z1345280375	23234	303.40	2.95	−4.90	2	5.2	72.76	−10.90	−43.03	96.8
Z1281686254	266430	431.54	4.51	−7.34	3	5.75	96.19	−11.20	−42.93	99.6
Z2003150479	565684	386.41	3.27	−7.21	2	6.25	123.68	−9.60	−42.59	98.0
Z5759799565	190665	417.26	2.24	−4.15	3	5.75	127.21	−11.24	−42.42	92.8
Z7693947094	1840737	370.33	3.33	−5.46	2	6.2	92.93	−11.01	−41.37	94.8
Z4595703814	4828297	394.48	3.53	−6.50	3	7	112.35	−9.67	−41.11	99.6
Z1262170288	235766	313.36	1.42	−4.35	4	5	134.30	−11.65	−40.71	99.6

*Note:* Reported values include MW, QPlogPo/w, QPlogS, donorHB/accptHB, PSA, Glide XP docking score, $∆G_{\text{bind}}$, and ALA433 hinge hydrogen bond occupancy (%). Compounds are listed in ascending order of $∆G_{\text{bind}}$.

### 2.5 In Vitro Assays Identify Four Active Anti‐ *T. brucei* Compounds

**FIGURE 4:**
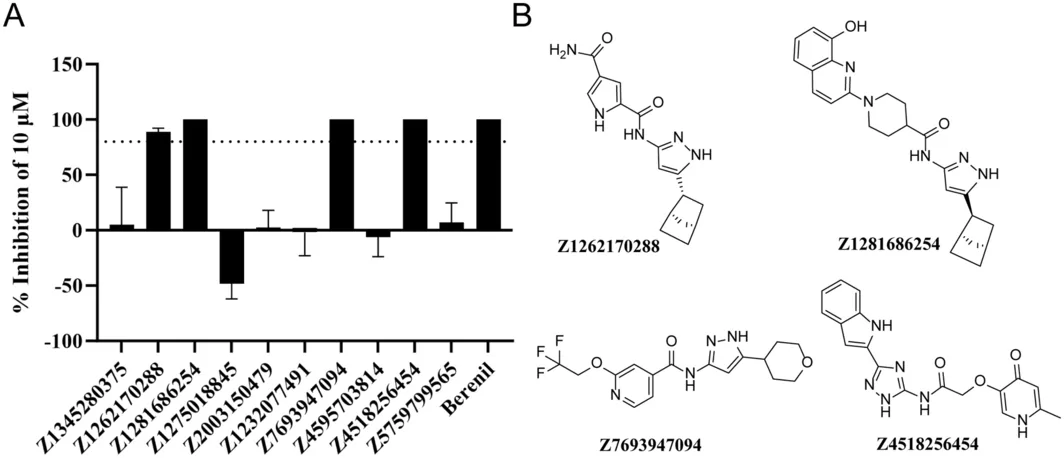
Growth inhibition screening of the 10 purchased compounds at 10 μM against the *T. brucei* bloodstream form. (A) Percent inhibition at 10 μM for each compound, with berenil included as a positive control. The dashed line marks the 80% inhibition cutoff used to select compounds for dose–response testing. (B) Chemical structures of the four compounds that achieved at least 80% inhibition at 10 μM.

**FIGURE 5:**
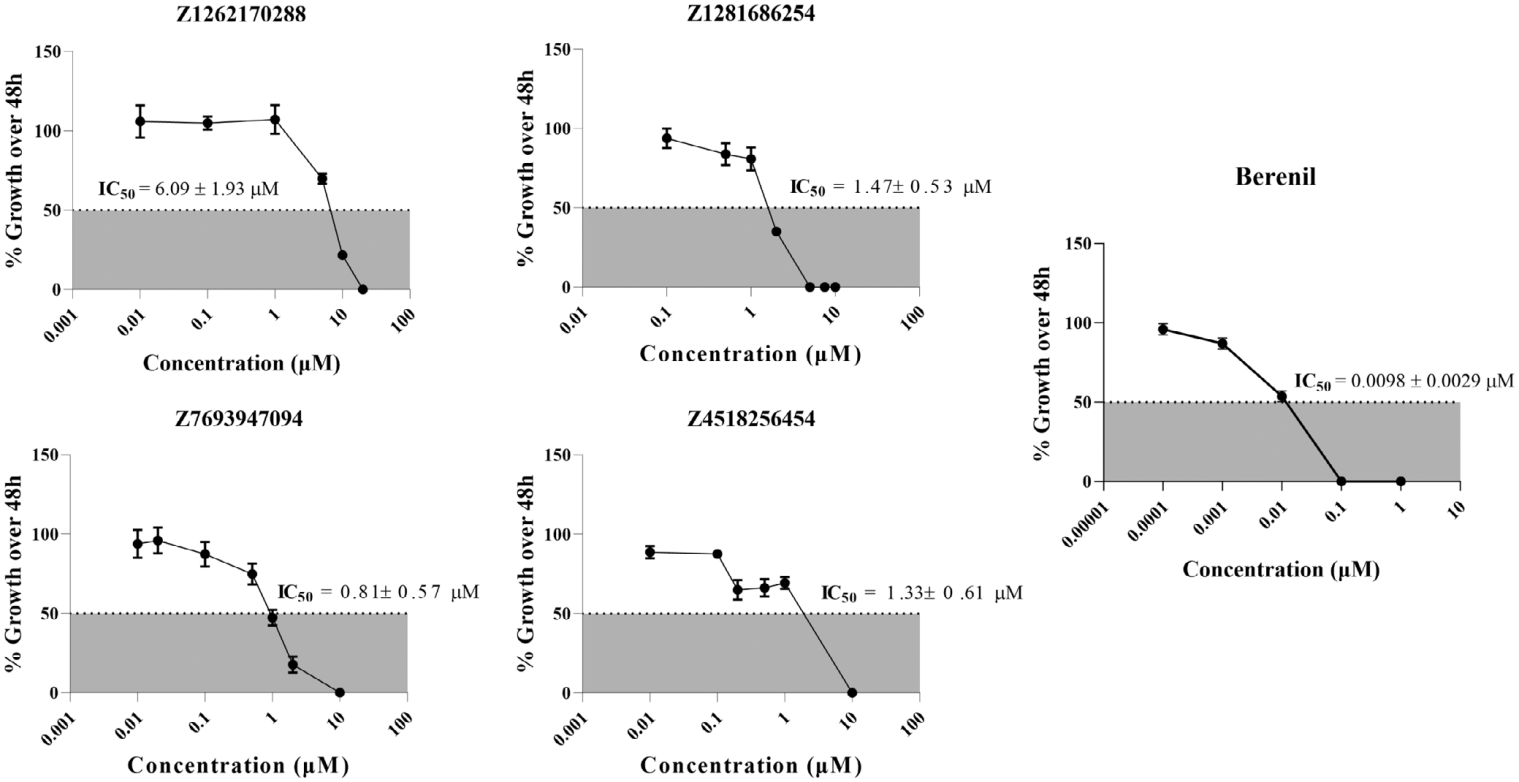
Concentration‐response curves for the four active compounds in the *T. brucei* growth inhibition assay over 48 h. IC_50_ values were obtained from three independent biological replicates and are reported as mean ± SEM.

To experimentally validate the computationally prioritized compounds, we evaluated all 10 candidates in the bloodstream form *T. brucei* growth inhibition assay, with berenil included as a positive control. Four compounds (Z1262170288, Z1281686254, Z7693947094, and Z4518256454) produced at least 80% inhibition at 10 μM, whereas the remaining compounds showed weaker activity (Figure 4). As shown in Figure 5, the IC_50_ values were 6.09 μM for Z1262170288, 1.47 μM for Z1281686254, 0.81 μM for Z7693947094, and 1.33 μM for Z4518256454. Three of the four active compounds, therefore, showed low‐micromolar potency, with Z7693947094 being the most potent in this set. The identification of four active compounds among the 10 tested candidates represents a favorable enrichment outcome for this data‐limited target.

**FIGURE 6:**
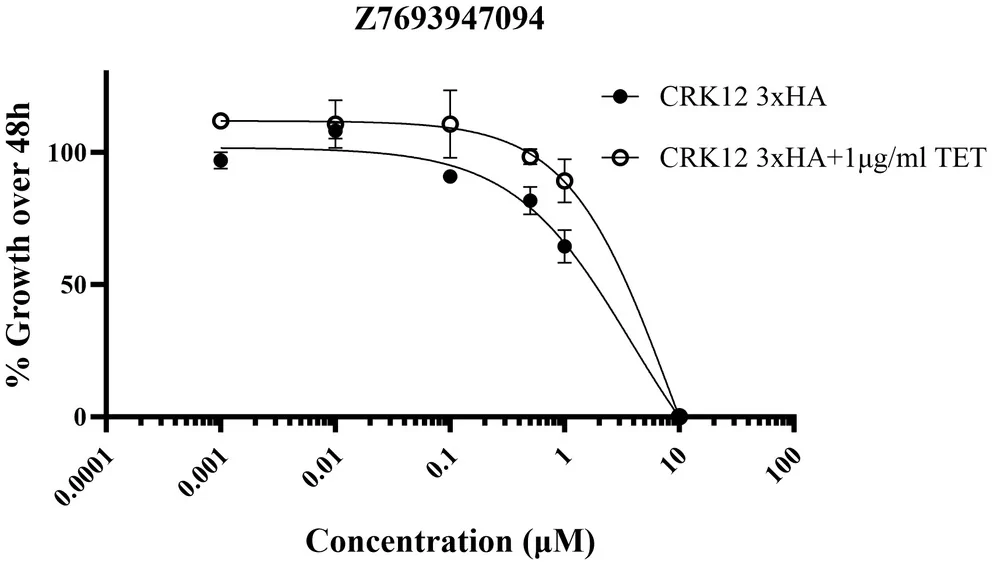
Effects of overexpression of CRK12 in *T. brucei* on the potency of Z7693947094.

After generating the inducible CRK12‐overexpressing strain, Western blot analysis confirmed the successful overexpression of CRK12 upon tetracycline induction (Figure S2A). Furthermore, the 7‐day cumulative growth curves showed comparable growth profiles for the strain cultured with and without tetracycline under the conditions tested (Figure S2B). Therefore, this strain was used for subsequent experiments. To assess potential CRK12‐related activity, compound Z7693947094 was further tested in an inducible CRK12‐overexpressing *T. brucei* line (Figure 6). CRK12 overexpression was associated with a modest rightward shift in the dose–response curve, corresponding to an approximately 2.2‐fold increase in the apparent IC_50_ value (from 3.83 to 8.32 μM). This shift was consistent with reduced cellular sensitivity following CRK12 overexpression and with possible CRK12‐related activity. However, the modest magnitude of the shift does not provide definitive evidence of direct on‐target engagement. Together, these results identify multiple compounds from the computationally prioritized set and motivate subsequent binding‐mode analysis.

### 2.6 Binding Mode Analysis of the Four Active Compounds

**FIGURE 7:**
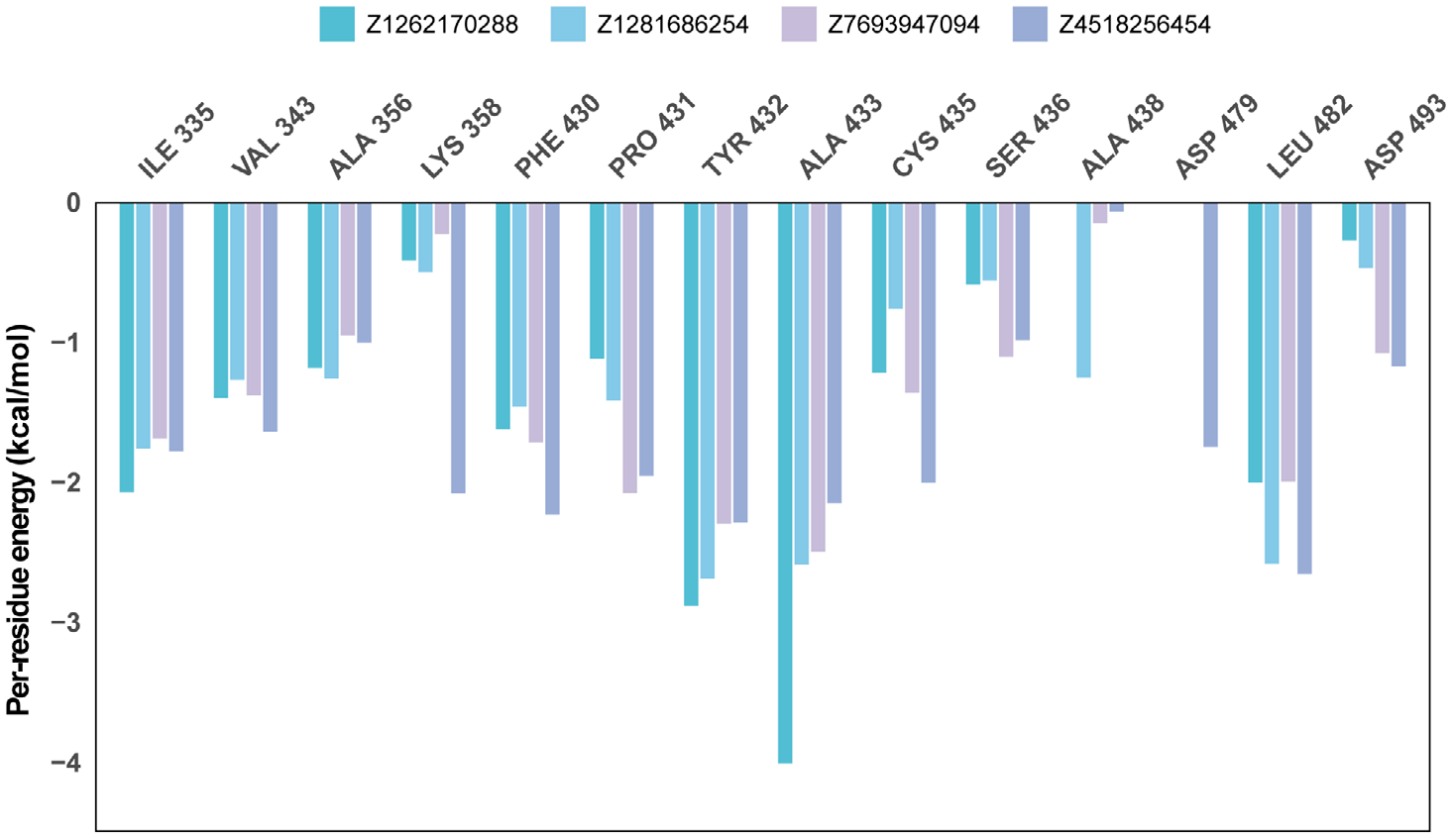
MM‐GBSA per‐residue energy decomposition for the CRK12‐ligand complexes of Z1262170288, Z1281686254, Z7693947094, and Z4518256454.

**FIGURE 8:**
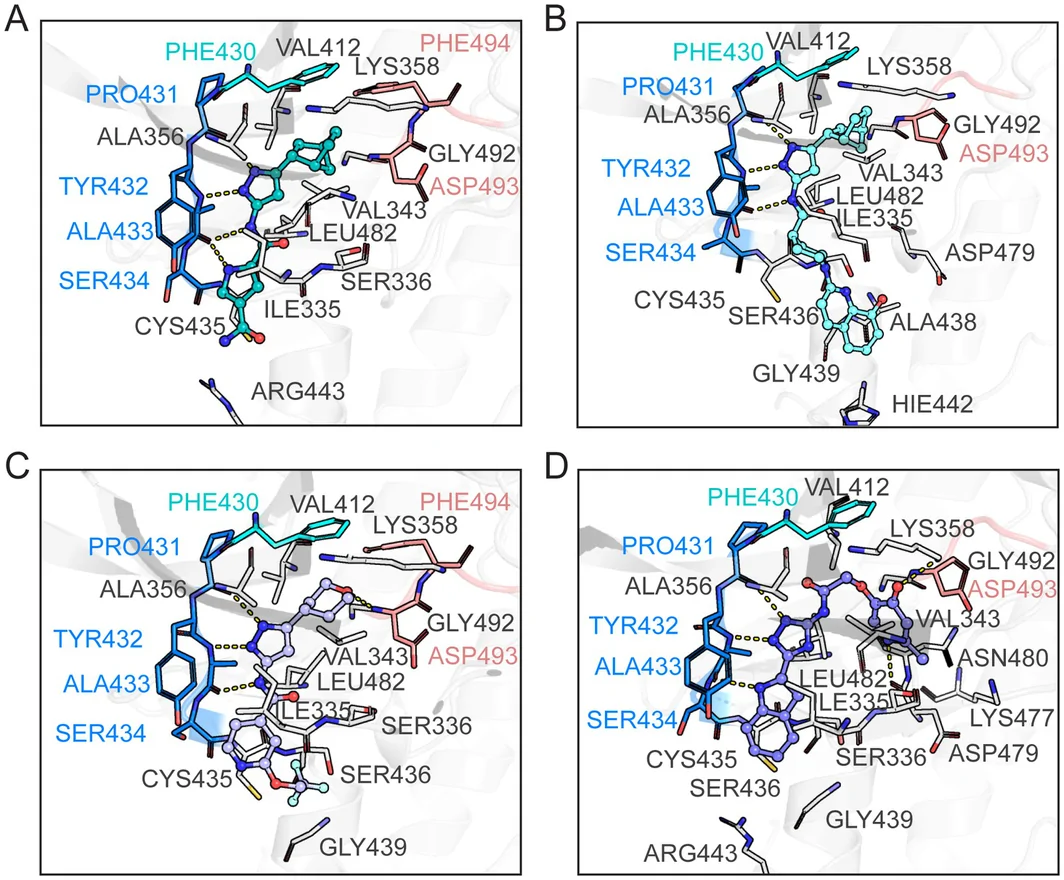
Representative binding modes of Z1262170288 (A), Z1281686254 (B), Z7693947094 (C), and Z4518256454 (D) in the CRK12 ATP‐binding site. Representative structures were obtained by clustering the MD trajectories. Ligands are shown as sticks, and key residues are shown as sticks and labeled. The CRK12 protein is shown as a gray cartoon. The gatekeeper residue is highlighted in cyan, hinge residues are highlighted in blue, and the DFG motif is highlighted in pink. Hydrogen bonds are shown as yellow dashed lines.

To rationalize the binding modes of the four active compounds, we analyzed the MD trajectories and representative conformations obtained through clustering. MM‐GBSA binding free energy estimates for the four active compounds fall within a similar range, from −44.82 to −40.71 kcal/mol (Table S2). Per‐residue decomposition further highlights consistently favorable contributions from the hinge region, including ALA433 and adjacent residues, which is in agreement with the hinge‐centered hydrogen bond pattern observed in Table 3 (Figure 7). Representative binding modes for Z1262170288, Z1281686254, Z7693947094, and Z4518256454 are shown in Figure 8. In all cases, the ligands occupy the adenine region of the ATP‐binding pocket and adopt a similar hinge‐anchored orientation, with the heteroaromatic core positioned to engage the hinge segment. We then quantified hydrogen bond occupancies between CRK12 and each active compound to assess interaction stability (Table 3). All four compounds formed high‐occupancy hydrogen bonds with the hinge region, primarily involving the backbone atoms of ALA433 and PRO431. This interaction pattern is consistent with the hinge‐directed binding mode proposed for CRK12 (Li et al. 2025; Smith et al. 2022) and resembles the hinge recognition observed for many human kinase inhibitors (Wu et al. 2015). Z1262170288 showed hinge hydrogen bonding dominated by ALA433, with occupancies in the 95.2%–99.6% range, and a hydrogen bond with PRO431 at 69.6%. Z1281686254 maintained a stable hinge hydrogen bond network involving ALA433 and PRO431. Z7693947094 retained high‐occupancy hinge hydrogen bonding and additionally formed a frequent hydrogen bond with ASP493. Z4518256454 exhibited high‐occupancy hinge hydrogen bonds together with a high‐occupancy hydrogen bond to ASP479, while interactions involving LYS358 were of lower occupancy. These results indicate stable hinge recognition across all four active compounds, together with additional contacts that differ among compounds.

**TABLE 3:** The hydrogen bond occupancies (%) between CRK12 and four active compounds during the simulations.

Vendor ID	Donor	Acceptor	Distance (Å)	Angle (°)	Occupancy (%)
Z1262170288	**ALA433@N‐H**	**LIGAND@N4**	2.94	160.09	99.6
	**LIGAND@N2‐H4**	**ALA433@O**	2.92	153.04	98.8
	**LIGAND@N3‐H6**	**ALA433@O**	3.14	157.67	95.2
	**LIGAND@N5‐H7**	**PRO431@O**	3.03	149.00	69.6
Z1281686254	**LIGAND@N3‐H14**	**ALA433@O**	2.94	162.82	99.6
	**ALA433@N‐H**	**LIGAND@N1**	3.00	155.42	95.6
	**LIGAND@N2‐H13**	**PRO431@O**	3.00	149.04	95.2
Z7693947094	**LIGAND@N4‐H7**	**PRO431@O**	2.93	158.50	98.8
	**LIGAND@N2‐H6**	**ALA433@O**	3.06	155.28	94.8
	**ALA433@N‐H**	**LIGAND@N3**	2.97	154.15	92.4
	ASP493@N‐H	LIGAND@O3	3.03	146.68	84.4
Z4518256454	LIGAND@N1‐H6	ASP479@O	2.94	162.6	99.6
	**ALA433@N‐H**	**LIGAND@N4**	3.03	157.84	98.8
	**LIGAND@N3‐H16**	**PRO431@O**	3.00	152.73	97.2
	**LIGAND@N6‐H10**	**ALA433@O**	2.95	142.86	94.8
	LYS358@NZ‐HZ3	LIGAND@O1	2.94	150.9	32.0
	LYS358@NZ‐HZ1	LIGAND@O1	2.97	147.41	21.6

*Note:* Hydrogen bonds involving the hinge residues ALA433 and PRO431 are highlighted in bold.

Three of the active compounds (Z1262170288, Z1281686254, and Z7693947094) share a 3‐amide‐substituted pyrazole as the hinge‐binding motif, whereas Z4518256454 contains a 1,2,4‐triazole motif (Figure 8). Despite this chemotype difference, all four active compounds adopt a similar hinge‐anchored pose in the adenine region of the ATP‐binding pocket and form multiple hydrogen bonds to the hinge backbone. In the pyrazole series, the pyrazole nitrogen provides a hydrogen bond acceptor site, and the pyrazole N‐H can act as a donor, while the 3‐amide contributes an additional N‐H donor, together supporting hinge hydrogen bonds with the ALA433 and PRO431 backbone (Figure 8A–C). In Z4518256454, the triazole ring provides hinge acceptor and donor functionalities, and the linked indole N‐H provides an additional donor, giving an analogous hinge hydrogen bonding pattern (Figure 8D). Beyond the hinge region, the four active compounds differ in substituents that extend into deeper pocket regions and toward the solvent‐exposed region, which introduce additional hydrogen bond contacts and distinguish their binding modes. Z1262170288 and Z1281686254 share the same norbornane‐derived substituent in the deep pocket and show highly similar poses around the hinge‐binding motif. Their main difference is in the solvent‐exposed substituent, where Z1262170288 carries a pyrrole group bearing a free amide oriented toward solvent, whereas Z1281686254 extends through a piperidine linker to a hydroxy‐substituted quinoline ring that is solvent‐exposed. Z7693947094 replaces the norbornane group with a tetrahydropyran substituent positioned in a comparable deep‐pocket region. The ring oxygen provides an additional acceptor site that forms a hydrogen bond with ASP493 in the DFG region, while a trifluoroethyl‐oxy substituted pyridine is oriented toward the solvent. In contrast, Z4518256454 contains an oxacetamide‐linked methyl‐pyridone that extends into a nearby subpocket. The pyridone carbonyl oxygen can form a hydrogen bond with LYS358, and the pyridone ring nitrogen forms a hydrogen bond with ASP479, with the indole moiety partially solvent‐exposed. Overall, the four active compounds share a hinge‐anchored binding mode, with differences in substituents that extend into deeper pocket regions and the solvent‐exposed regions. These analyses support the proposed binding modes for the active compounds and underscore the potential of these chemotypes as starting points for hit‐to‐lead optimization.

## 3 Methods and Materials

### 3.1 Compound Library and the Benchmark Dataset

The screening library was the TargetMol collection provided as SDF files containing ~8.6 million compounds. The CRK12 active/decoy benchmark, comprising active compounds and decoys generated using DUD‐E (Mysinger et al. 2012) at a ratio of 1:50, was adopted from our previously published work (Li et al. 2025) to evaluate the performance of virtual screening methods.

### 3.2 CRK12 Modeling and Receptor Selection

#### 3.2.1 CRK12 Modeling and MD Trajectory Source

In the absence of an experimentally resolved *T. brucei* CRK12 structure, an AlphaFold2‐predicted CRK12 structure (Jumper et al. 2021) was used as the starting receptor for CRK12 modeling. Model confidence and stereochemical quality were assessed in a previous study (Li et al. 2025). The ATP‐binding pocket was identified using SiteMap in Schrödinger (Release 2022, Schrödinger LLC, New York, NY, USA). The reference ligand cmpd2 was docked into the ATP site, and the resulting CRK12‐cmpd2 complex was refined by a long‐timescale molecular dynamics simulation (500 ns) following our previous protocol. The CRK12‐cmpd2 trajectory served as the conformational ensemble for receptor selection in the present study.

#### 3.2.2 Pocket‐Focused Clustering

Pocket‐focused clustering was performed using cpptraj in AMBER20 on frames from the equilibrated region of the MD trajectory. Pocket residues were defined in PyMOL as residues with any heavy atom within 6 Å of cmpd2 in the starting CRK12‐cmpd2 complex. The RMSD of heavy atoms of the predefined pocket residues was used for hierarchical clustering to identify the most populated pocket states. The most populated cluster centroids were retained as candidate receptor conformations.

#### 3.2.3 CRK12 Receptor Selection

The MD‐refined receptor conformation used previously (“Refined”) and the pocket‐derived centroids (Rep C0‐C4) were benchmarked using Glide Standard Precision (SP) and Extra Precision (XP) docking in Schrödinger. Docking grids were generated consistently for each receptor by centering on the cmpd2 pose in the ATP‐binding site, and identical docking settings were applied across receptors. Screening performance was assessed on the established active/decoy benchmark using the area under the receiver operating characteristic curve (ROC‐AUC) and enrichment factors at early cutoffs (EF1%, EF5%, and EF10%) (Halgren et al. 2004). The receptor conformation for prospective docking was selected by jointly considering ROC‐AUC and early enrichment.

### 3.3 Fingerprint‐Based Similarity Screening

The reference ligand cmpd2 was used as the query structure for fingerprint‐based screening. Four fingerprints (Linear, MACCS, Radial, and MolPrint2D) were generated using Schrödinger Canvas, and similarity was computed using the Tanimoto coefficient (Stumpfe and Bajorath 2011). Retrospective performance was assessed on the established active/decoy benchmark using ROC‐AUC and early enrichment factors. MACCS fingerprint screening was applied prospectively to the TargetMol library using a fixed similarity threshold (Tanimoto ≥ 0.60), and retrieved molecules were retained for downstream screening.

### 3.4 Complex‐Based Pharmacophore Screening

Before pharmacophore screening, the ligands were prepared using Schrödinger LigPrep. The complex‐based pharmacophore model was constructed from a representative MD‐refined CRK12‐cmpd2 complex (Rep C0) using Phase. Based on the representative CRK12‐cmpd2 binding pose, pharmacophore features corresponding to the hinge‐region hydrogen bonding pattern and the aminothiazole aromatic ring were manually selected to define the final hypothesis. The selected ADDR hypothesis contained one hydrogen bond acceptor feature, two hydrogen bond donor features, and one aromatic ring feature, labeled as A1, D6, D8, and R15, respectively. Specifically, A1 was mapped to the ring nitrogen atom of the aminothiazole moiety, D8 to the exocyclic primary amino group attached to the aminothiazole ring, D6 to the secondary amino linker connecting the cyclohexyl group to the aminothiazole ring, and R15 to the centroid and orientation of the aminothiazole ring. The hydrogen bond donor and acceptor features used the default Phase projection definitions, whereas the aromatic ring feature was defined by the centroid and orientation vector of the aminothiazole ring. A default feature‐matching tolerance radius of 2.0 Å was applied to all four features. A receptor‐based excluded volume shell was included in the pharmacophore model using the default Phase settings. The excluded volumes were defined using the van der Waals radii of receptor atoms with a fixed scaling factor of 0.50. Receptor atoms whose surfaces were within 2.0 Å of the ligand surface were ignored, and the excluded‐volume shell thickness was limited to 5.0 Å. The TargetMol library was screened using Schrödinger Phase, and pharmacophore matching required at least two of the four features to be satisfied. Matched conformations were ranked by Phase score. The top 300,000 conformations were retained and deduplicated to yield unique compounds. The pharmacophore‐selected compounds were combined with the MACCS fingerprint hits by a union operation to generate the union set for structure‐based screening.

### 3.5 Structure‐Based Virtual Screening

#### 3.5.1 Ligand Preparation

All compounds used in structure‐based steps, including benchmark docking and docking of the prescreened union set, were prepared using Schrödinger LigPrep. Ionization states were generated at pH 7.0 ± 2.0 using Epik. Desalting and tautomer generation were enabled, and stereoisomers were enumerated up to 32 per compound while retaining specified chiralities.

#### 3.5.2 Grid Generation and Staged Docking

The union set was subjected to Lipinski's rule‐of‐five filtering (Lipinski et al. 2001) with the relevant descriptors calculated using QikProp (Ioakimidis et al. 2008) and reactive‐group removal. Using the selected receptor conformation, the filtered library was then screened through a staged Glide docking workflow (Glide HTVS 10%, SP 30%, XP top 5000). The top 5000 XP‐ranked compounds were retained for post‐docking prioritization. The XP‐ranked compounds were clustered into 100 groups using Canvas with K‐means clustering, and representative compounds were selected based on docking rank, cluster representation, and visual inspection of the predicted binding modes, with attention to hinge‐region hydrogen bonding and the absence of severe steric clashes.

### 3.6 MD‐Based Refinement and MM‐GBSA Binding Free Energy Estimation

MD simulations of the 40 CRK12‐ligand complexes were performed with the AMBER20 package (University of California, San Francisco, CA). The protein was parameterized with the AMBER ff14SB force field (Maier et al. 2015). Ligands were parameterized with GAFF (Wang et al. 2004), and AM1‐BCC (Jakalian et al. 2002) partial charges were assigned using antechamber. Each complex was solvated in a TIP3P (Price and Brooks 2004) water box extending 10 Å from the solute. The system was neutralized with sodium and chloride ions and simulated under periodic boundary conditions. Long‐range electrostatics were treated with particle‐mesh Ewald (PME) (Toukmaji et al. 2000), and bonds involving hydrogen atoms were constrained using SHAKE (Miyamoto and Kollman 1992). Systems were minimized using 2500 steps of steepest‐descent minimization followed by 2500 steps of conjugate‐gradient minimization, heated from 0 to 300 K under NVT using a Langevin thermostat with harmonic restraints on the complex (2.0 kcal·mol^−1^·Å^−2^), and equilibrated under NPT at 300 K with restraints gradually reduced from 2.0 to 0 kcal·mol^−1^·Å^−2^. Production simulations were then performed for 30 ns under NPT at 300 K without any restraints. Hydrogen bond occupancy was quantified using a donor‐acceptor distance cutoff of 3.5 Å and an angle cutoff of 120°. Molecular mechanics‐generalized Born surface area (MM‐GBSA) (Wang et al. 2019) binding free energies were estimated from snapshots extracted from the final 5 ns of each trajectory without considering entropy.

### 3.7 In Vitro Assays

#### 3.7.1 Trypanosome Culture and In Vitro Growth Inhibition Assay

Pleomorphic bloodstream form *T. brucei* AnTat1.1 parasites were cultured in HMI‐9 medium (Hirumi and Hirumi 1989) as previously described (Delauw et al. 1985). Trypanosomes were maintained at densities below 1 × 10^6^ cells/mL at 37°C with 5% CO_2_. For compound testing, parasites were seeded at 5 × 10^3^ cells/mL in 24‐well plates and treated with the indicated compound concentrations. Parasite growth was assessed after 48 h of incubation by counting motile trypanosomes using a Neubauer hemocytometer under light microscopy. Concentration‐response curves were generated using GraphPad Prism software, and IC_50_ values are reported as mean ± SEM from at least three independent biological replicates.

#### 3.7.2 CRK12 Overexpression in *T. brucei*

To generate an inducible CRK12‐overexpressing *T. brucei* bloodstream form cell line, wild‐type cells were first transfected with the pSMOX plasmid encoding T7 RNA polymerase and tetracycline repressor (TetR) to generate a pSMOX single‐marker cell line, as previously described (Poon et al. 2012). Subsequently, the CRK12 coding sequence (TriTrypDB Tb927.11.12310) fused to a C‐terminal 3 × HA tag was cloned into the pDex577 vector for tetracycline‐inducible overexpression (Kelly et al. 2007). The resulting plasmid (pDex577‐CRK12‐3 × HA) was linearized with NotI and transfected into the pSMOX‐expressing cell line as described above. Transfectants were selected with 2.5 μg/mL phleomycin, and single clones were obtained by limiting dilution. To verify the successful overexpression of CRK12, samples were collected after induction with 1 μg/mL tetracycline for 24 h and subjected to qualitative Western blot analysis using an anti‐HA antibody, with β‐tubulin used as the loading control. Subsequently, 7‐day cumulative growth curves were generated to evaluate the effect of tetracycline‐induced CRK12 overexpression on strain growth. The validated clone was treated with both tetracycline and the test compound for sensitivity assays.

## 4 Conclusions

This study presents an integrated CRK12‐guided virtual screening and experimental validation workflow for identifying anti‐*Trypanosoma brucei* chemical matter under the conditions of limited ligand data and no resolved target structure. An AlphaFold2‐derived CRK12 model refined by long‐timescale MD was used to derive a receptor ensemble, and pocket‐focused clustering with retrospective benchmarking supported selection of a receptor conformation for prospective docking. By combining complementary ligand‐based prescreening strategies, staged docking, and MD‐based refinement, we efficiently reduced a multi‐million‐compound library to a compact experimental set. Among the 10 purchased compounds, four showed strong growth inhibition against *T. brucei* at 10 μM, with IC_50_ values of 6.09, 1.47, 0.81, and 1.33 μM. Three of these compounds displayed low‐micromolar potency. This hit rate supports the practical enrichment achieved by the stepwise computational prioritization. A CRK12‐overexpression sensitivity assay for the most potent hit produced a modest shift under the current conditions, indicating that additional target‐engagement evidence will be required to establish on‐target activity in cells. The binding‐mode analysis of the active compounds consistently suggested hinge‐directed anchoring in the ATP‐binding pocket. Hydrogen bond occupancy analysis highlighted persistent hinge interactions, and per‐residue energy decomposition indicated favorable contributions from hinge‐proximal residues, providing a structural rationale for the proposed CRK12 binding modes and guidance for follow‐up analogue design.

## Author Contributions

**Chenggong Fu:** validation, formal analysis, data curation, visualization, writing – original draft. **Lingling Wang:** visualization. **Jiayi Luo:** methodology, validation, investigation. **Dehua Lai:** resources, writing – review and editing, supervision. **Xiaomeng Liu:** formal analysis. **Henry H. Y. Tong:** resources. **Qin Li:** conceptualization, methodology, investigation, data curation, formal analysis, visualization, writing – original draft. **Xiaojun Yao:** writing – review and editing. **Xiaoqing Gong:** methodology. **Qianqian Zhang:** methodology, formal analysis. **Huanxiang Liu:** conceptualization, writing – review and editing, funding acquisition, project administration, supervision, resources. **Jiayue Qiu:** formal analysis.

## Funding

This work was supported by Macao Science and Technology Development Fund, 0012/2025/ASJ, 0043/2023/AFJ; National Natural Science Foundation of China, 32270446.

## Conflicts of Interest

The authors declare no conflicts of interest.

## Supporting information


**Table S1:** Summary MD‐based refinement results for the 40 CRK12‐ligand complexes simulated for 30 ns, including Glide XP docking score, MM‐GBSA binding free energy estimate (ΔG), and ALA433 hinge H‐bond occupancy (%). Compounds are ordered by $∆G_{\text{bind}}$ in ascending order. The 11 highest‐ranked compounds were prioritized for purchase. Internal ID 176262 was not commercially available, and the remaining 10 compounds were purchased for biological testing and are shown in bold.
**Table S2:** MM‐GBSA binding free energy estimates and energy components (kcal/mol) for the 10 purchased compounds. Energies are ordered by $∆G_{\text{bind}}$ in ascending order.
**Table S3:** Hydrogen‐bond occupancy (%) between the CRK12 hinge residue ALA433 and each of the 10 purchased compounds. Entries are ordered by $∆G_{\text{bind}}$ ordering as Table 2.
**Figure S1:** The RMSD of the heavy atoms of the pocket and ligand for the 40 CRK12‐ligand complexes during 30 ns MD simulations.
**Figure S2:** (A) CRK12 overexpression was induced with tetracycline (1 μg/mL, 24 h) and confirmed by Western blot analysis using an anti‐HA antibody. β‐Tubulin was used as the loading control. (B) Seven‐day cumulative growth curves of the inducible CRK12‐overexpressing cell line cultured with or without 1 μg/mL tetracycline.

## Data Availability

The data that support the findings of this study are available on request from the corresponding author. The data are not publicly available due to privacy or ethical restrictions.
