# Supplementary material for: Identification of New Anti‐Trypanosoma brucei Leads: An Integrated CRK12‐Guided Virtual Screen With Experimental Validation
Source: Chem Biol Drug Des. 2026 Aug 30;108(3):e70389. doi: 10.1111/cbdd.70389 (PMC13526909; doi:10.1111/cbdd.70389)
Supplement: Supplementary file 1 [file CBDD-108-e70389-s001.docx]

Supporting Information

**Identification of New Anti-Trypanosoma brucei Leads: An Integrated CRK12-Guided Virtual Screen with Experimental Validation**

Qin Li^a&^, Chenggong Fu^a&^, Jiayi Luo^b^, Qianqian Zhang^a^, Lingling Wang^a^, Xiaomeng Liu^a^, Jiayue Qiu^a^, Xiaoqing Gong^a^, Henry H. Y. Tong^a^, Xiaojun Yao^a^, Dehua Lai^b*^, Huanxiang Liu^a*^

^a^Faculty of Applied Sciences, Macao Polytechnic University, Macao SAR, The People's Republic of China.

^b^MOE Key Laboratory of Gene Function and Regulation, State Key Laboratory of Biocontrol and Guangdong Provincial Key Laboratory of Aquatic Economic Animals, School of Life Sciences, Sun Yat-Sen University, Guangzhou 510275, The People's Republic of China.

&These authors contributed equally to this work.

Table S1. Summary MD-based refinement results for the 40 CRK12-ligand complexes simulated for 30 ns, including Glide XP docking score, MM-GBSA binding free energy estimate (ΔG), and ALA433 hinge H-bond occupancy (%). Compounds are ordered by $\text{∆G}_{bind}$ in ascending order. The eleven highest-ranked compounds were prioritized for purchase. Internal ID 176262 was not commercially available, and the remaining ten compounds were purchased for biological testing and are shown in bold.

| Internal ID | Docking score | $\text{∆G}_{bind}$ | Hinge H-bond occupancy (%) |
| --- | --- | --- | --- |
| **7431599** | -11.41 | -44.82 | 98.80 |
| **560407** | -9.12 | -43.57 | 92.80 |
| **1206270** | -10.65 | -43.11 | 97.60 |
| **23234** | -10.89 | -43.03 | 96.80 |
| **266430** | -11.2 | -42.93 | 99.60 |
| **565684** | -9.61 | -42.59 | 98.00 |
| **190665** | -11.24 | -42.42 | 92.80 |
| **1840737** | -11.01 | -41.37 | 94.80 |
| 176262 | -10.63 | -41.25 | 97.60 |
| **4828297** | -9.67 | -41.11 | 99.60 |
| **235766** | -11.65 | -40.71 | 99.60 |
| 19415 | -11.07 | -39.84 | 99.60 |
| 5386858 | -11.38 | -39.68 | 98.00 |
| 1542383 | -11.36 | -39.24 | 87.20 |
| 5825281 | -11.08 | -38.89 | 99.60 |
| 761120 | -10.64 | -38.60 | 96.40 |
| 7699011 | -11.98 | -38.38 | 80.00 |
| 700944 | -11.36 | -38.26 | 99.60 |
| 4908008 | -11.17 | -38.25 | 89.60 |
| 211662 | -11.42 | -37.52 | 99.20 |
| 5357547 | -11.69 | -37.31 | 96.40 |
| 162830 | -11.35 | -36.97 | 94.80 |
| 754631 | -11.3 | -36.10 | 98.00 |
| 6885676 | -10.67 | -35.36 | 94.40 |
| 249873 | -11.32 | -34.09 | 94.00 |
| 527476 | -10.07 | -33.93 | 99.20 |
| 774084 | -10.59 | -33.76 | 99.60 |
| 671121 | -10.42 | -33.69 | 82.40 |
| 1637553 | -10.78 | -33.37 | 95.60 |
| 646212 | -11.4 | -33.14 | 97.20 |
| 7915767 | -10.25 | -33.10 | 88.40 |
| 6889905 | -11.47 | -32.64 | 97.60 |
| 22947 | -10.62 | -31.05 | 95.60 |
| 7380705 | -11.97 | -31.04 | 93.20 |
| 759014 | -11.38 | -30.25 | 98.00 |
| 7455992 | -11.43 | -30.06 | 95.60 |
| 1662620 | -10.41 | -29.50 | 87.60 |
| 5257285 | -10.68 | -26.99 | 72.80 |
| 788579 | -10.31 | -25.33 | 95.20 |
| 4746418 | -10.43 | -21.76 | 0.00 |

Table S2. MM-GBSA binding free energy estimates and energy components (kcal/mol) for the 10 purchased compounds. Energies are ordered by $\text{∆G}_{bind}$ in ascending order.

| Vendor ID | Internal ID | $\text{∆E}_{VDW}$ | $\text{∆E}_{ELE}$ | $\text{∆E}_{GB}$ | $\text{∆E}_{SURF}$ | $\text{∆G}_{GAS}$ | $\text{∆G}_{SOLV}$ | $\text{∆G}_{bind}$ |
| --- | --- | --- | --- | --- | --- | --- | --- | --- |
| Z4518256454 | 7431599 | -50.61 | -42.10 | 53.87 | -5.98 | -92.71 | 47.89 | -44.82 |
| Z1275018845 | 560407 | -47.64 | -20.68 | 30.21 | -5.46 | -68.32 | 24.75 | -43.57 |
| Z1232077491 | 1206270 | -47.02 | -39.44 | 49.28 | -5.94 | -86.46 | 43.34 | -43.11 |
| Z1345280375 | 23234 | -41.21 | -19.58 | 22.61 | -4.85 | -60.79 | 17.76 | -43.03 |
| Z1281686254 | 266430 | -46.88 | -14.97 | 24.22 | -5.29 | -61.85 | 18.93 | -42.93 |
| Z2003150479 | 565684 | -50.42 | -13.59 | 27.45 | -6.03 | -64.01 | 21.42 | -42.59 |
| Z5759799565 | 190665 | -48.84 | -26.09 | 38.35 | -5.85 | -74.93 | 32.5 | -42.42 |
| Z7693947094 | 1840737 | -43.58 | -22.20 | 29.93 | -5.52 | -65.78 | 24.41 | -41.37 |
| Z4595703814 | 4828297 | -47.11 | -35.94 | 47.94 | -5.99 | -83.05 | 41.95 | -41.11 |
| Z1262170288 | 235766 | -41.18 | -23.54 | 28.68 | -4.67 | -64.72 | 24.01 | -40.71 |

Table S3. Hydrogen-bond occupancy (%) between the CRK12 hinge residue ALA433 and each of the 10 purchased compounds. Entries are ordered by $\text{∆G}_{bind}$ ordering as Table 2.

| Vendor ID | Internal ID | Donor | Accepter | Occupancy (%) |
| --- | --- | --- | --- | --- |
| Z4518256454 | 7431599 | ALA433@N-H | LIGAND@N4 | 98.8 |
|  |  | LIGAND@N6-H10 | ALA433@O | 94.8 |
| Z1275018845 | 560407 | ALA433@N-H | LIGAND@N4 | 88.0 |
|  |  | LIGAND@N3-H10 | ALA433@O | 92.8 |
| Z1232077491 | 1206270 | ALA433@N-H | LIGAND@N3 | 92.4 |
|  |  | LIGAND@N2-H9 | ALA433@O | 97.6 |
| Z1345280375 | 23234 | ALA433@N-H | LIGAND@N2 | 96.8 |
|  |  | LIGAND@N1-H12 | ALA433@O | 96.0 |
| Z1281686254 | 266430 | ALA433@N-H | LIGAND@N1 | 95.6 |
|  |  | LIGAND@N3-H14 | ALA433@O | 99.6 |
| Z2003150479 | 565684 | ALA433@N-H | LIGAND@N3 | 98.0 |
|  |  | LIGAND@N4-H7 | ALA433@O | 56.0­­ |
| Z5759799565 | 190665 | ALA433@N-H | LIGAND@N3 | 82.4 |
|  |  | LIGAND@N2-H13 | ALA433@O | 92.8 |
| Z7693947094 | 1840737 | ALA433@N-H | LIGAND@N3 | 92.4 |
|  |  | LIGAND@N2-H6 | ALA433@O | 94.8 |
| Z4595703814 | 4828297 | ALA433@N-H | LIGAND@O2 | 99.6 |
| Z1262170288 | 235766 | ALA433@N-H | LIGAND@N4 | 99.6 |
|  |  | LIGAND@N2-H4 | ALA433@O | 98.8 |
|  |  | LIGAND@N3-H6 | ALA433@O | 95.2 |

Figure S1. The RMSD of the heavy atoms of the pocket and ligand for the 40 CRK12-ligand complexes during 30 ns MD simulations.

Figure S2. (A) CRK12 overexpression was induced with tetracycline (1 μg/mL, 24 h) and confirmed by Western blot analysis using an anti-HA antibody. β-Tubulin was used as the loading control. (B) Seven-day cumulative growth curves of the inducible CRK12-overexpressing cell line cultured with or without 1 μg/mL tetracycline.
